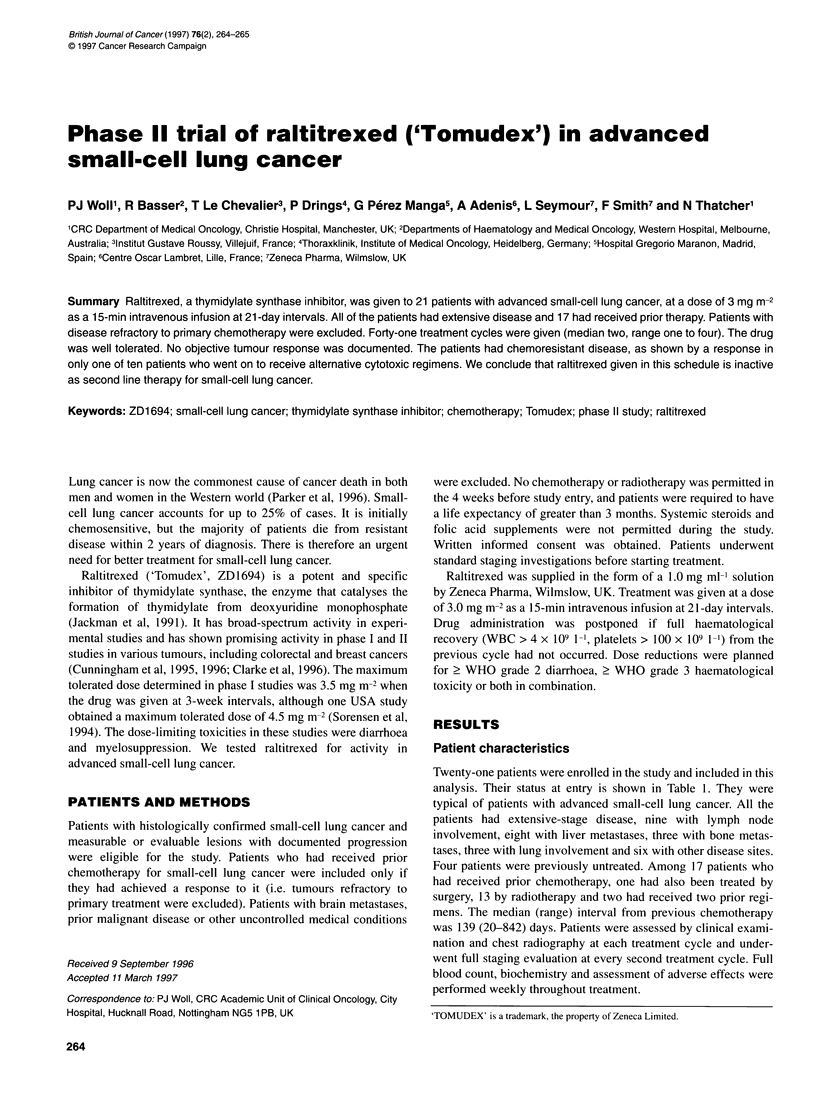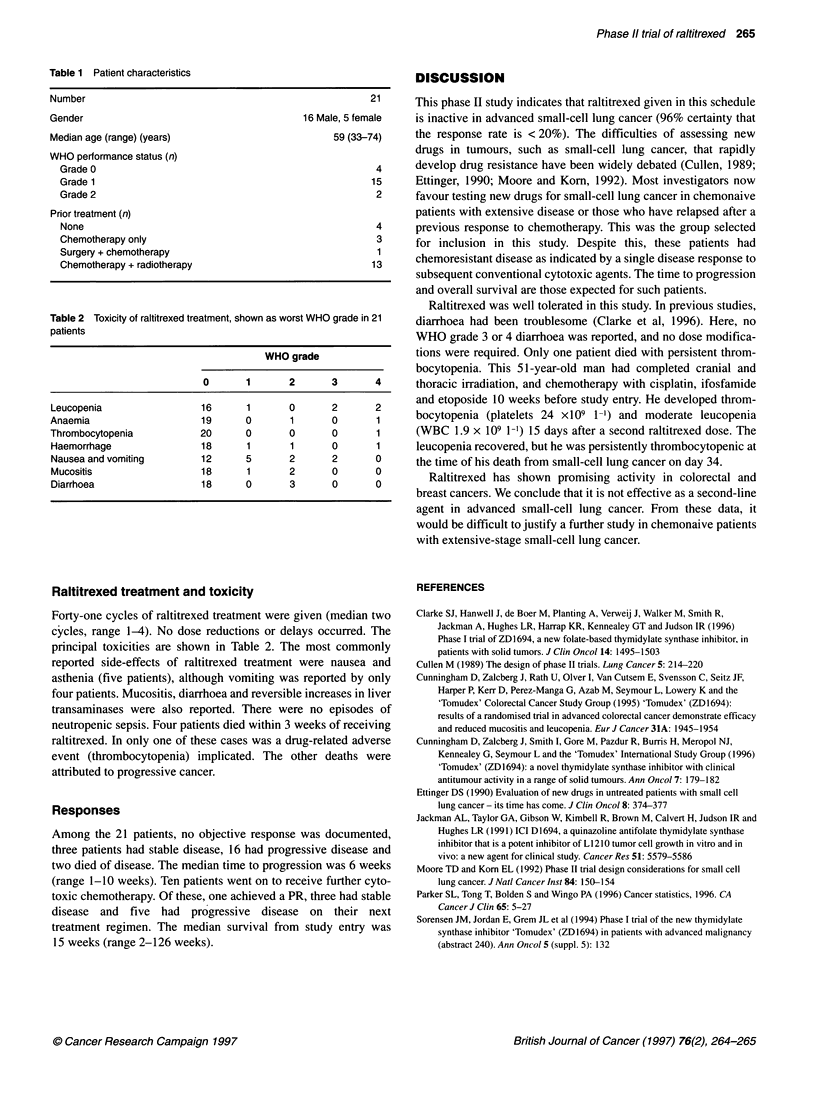# Phase II trial of raltitrexed ('Tomudex') in advanced small-cell lung cancer.

**DOI:** 10.1038/bjc.1997.373

**Published:** 1997

**Authors:** P. J. Woll, R. Basser, T. Le Chevalier, P. Drings, G. Perez Manga, A. Adenis, L. Seymour, F. Smith, N. Thatcher

**Affiliations:** CRC Department of Medical Oncology, Christie Hospital, Manchester, UK.

## Abstract

Raltitrexed, a thymidylate synthase inhibitor, was given to 21 patients with advanced small-cell lung cancer, at a dose of 3 mg m(-2) as a 15-min intravenous infusion at 21-day intervals. All of the patients had extensive disease and 17 had received prior therapy. Patients with disease refractory to primary chemotherapy were excluded. Forty-one treatment cycles were given (median two, range one to four). The drug was well tolerated. No objective tumour response was documented. The patients had chemoresistant disease, as shown by a response in only one of ten patients who went on to receive alternative cytotoxic regimens. We conclude that raltitrexed given in this schedule is inactive as second line therapy for small-cell lung cancer.


British Journal of Cancer (1997) 76(2), 264–265
© 1997 Cancer Research Campaign

### Phase II trial of raltitrexed ('Tomudex') in advanced small-cell lung cancer

**PJ Woll[1], R Basser[2], T Le Chevalier[3], P Drings[4], G Pérez Manga[5], A Adenis[6], L Seymour[7], F Smith[7] and N Thatcher[1]**

[1]CRC Department of Medical Oncology, Christie Hospital, Manchester, UK; [2]Departments of Haematology and Medical Oncology, Western Hospital, Melbourne, Australia; [3]Institut Gustave Roussy, Villejuif, France; [4]Thoraxklinik, Institute of Medical Oncology, Heidelberg, Germany; [5]Hospital Gregorio Maranon, Madrid, Spain; [6]Centre Oscar Lambret, Lille, France; [7]Zeneca Pharma, Wilmslow, UK

**Summary** Raltitrexed, a thymidylate synthase inhibitor, was given to 21 patients with advanced small-cell lung cancer, at a dose of 3 mg m$^{-2}$ as a 15-min intravenous infusion at 21-day intervals. All of the patients had extensive disease and 17 had received prior therapy. Patients with disease refractory to primary chemotherapy were excluded. Forty-one treatment cycles were given (median two, range one to four). The drug was well tolerated. No objective tumour response was documented. The patients had chemoresistant disease, as shown by a response in only one of ten patients who went on to receive alternative cytotoxic regimens. We conclude that raltitrexed given in this schedule is inactive as second line therapy for small-cell lung cancer.

**Keywords:** ZD1694; small-cell lung cancer; thymidylate synthase inhibitor; chemotherapy; Tomudex; phase II study; raltitrexed

Lung cancer is now the commonest cause of cancer death in both men and women in the Western world (Parker et al, 1996). Small-cell lung cancer accounts for up to 25% of cases. It is initially chemosensitive, but the majority of patients die from resistant disease within 2 years of diagnosis. There is therefore an urgent need for better treatment for small-cell lung cancer.

Raltitrexed ('Tomudex', ZD1694) is a potent and specific inhibitor of thymidylate synthase, the enzyme that catalyses the formation of thymidylate from deoxyuridine monophosphate (Jackman et al, 1991). It has broad-spectrum activity in experimental studies and has shown promising activity in phase I and II studies in various tumours, including colorectal and breast cancers (Cunningham et al, 1995, 1996; Clarke et al, 1996). The maximum tolerated dose determined in phase I studies was 3.5 mg m$^{-2}$ when the drug was given at 3-week intervals, although one USA study obtained a maximum tolerated dose of 4.5 mg m$^{-2}$ (Sorensen et al, 1994). The dose-limiting toxicities in these studies were diarrhoea and myelosuppression. We tested raltitrexed for activity in advanced small-cell lung cancer.

### PATIENTS AND METHODS

Patients with histologically confirmed small-cell lung cancer and measurable or evaluable lesions with documented progression were eligible for the study. Patients who had received prior chemotherapy for small-cell lung cancer were included only if they had achieved a response to it (i.e. tumours refractory to primary treatment were excluded). Patients with brain metastases, prior malignant disease or other uncontrolled medical conditions were excluded. No chemotherapy or radiotherapy was permitted in the 4 weeks before study entry, and patients were required to have a life expectancy of greater than 3 months. Systemic steroids and folic acid supplements were not permitted during the study. Written informed consent was obtained. Patients underwent standard staging investigations before starting treatment.

Raltitrexed was supplied in the form of a 1.0 mg ml$^{-1}$ solution by Zeneca Pharma, Wilmslow, UK. Treatment was given at a dose of 3.0 mg m$^{-2}$ as a 15-min intravenous infusion at 21-day intervals. Drug administration was postponed if full haematological recovery (WBC $> 4 \times 10^9$ l$^{-1}$, platelets $> 100 \times 10^9$ l$^{-1}$) from the previous cycle had not occurred. Dose reductions were planned for $\geq$ WHO grade 2 diarrhoea, $\geq$ WHO grade 3 haematological toxicity or both in combination.

### RESULTS

### Patient characteristics

Twenty-one patients were enrolled in the study and included in this analysis. Their status at entry is shown in Table 1. They were typical of patients with advanced small-cell lung cancer. All the patients had extensive-stage disease, nine with lymph node involvement, eight with liver metastases, three with bone metastases, three with lung involvement and six with other disease sites. Four patients were previously untreated. Among 17 patients who had received prior chemotherapy, one had also been treated by surgery, 13 by radiotherapy and two had received two prior regimens. The median (range) interval from previous chemotherapy was 139 (20–842) days. Patients were assessed by clinical examination and chest radiography at each treatment cycle and underwent full staging evaluation at every second treatment cycle. Full blood count, biochemistry and assessment of adverse effects were performed weekly throughout treatment.

*Received 9 September 1996*
*Accepted 11 March 1997*

*Correspondence to:* PJ Woll, CRC Academic Unit of Clinical Oncology, City Hospital, Hucknall Road, Nottingham NG5 1PB, UK

'TOMUDEX' is a trademark, the property of Zeneca Limited.

**Table 1** Patient characteristics

| | |
|---|---|
| Number | 21 |
| Gender | 16 Male, 5 female |
| Median age (range) (years) | 59 (33–74) |
| WHO performance status (*n*) | |
| Grade 0 | 4 |
| Grade 1 | 15 |
| Grade 2 | 2 |
| Prior treatment (*n*) | |
| None | 4 |
| Chemotherapy only | 3 |
| Surgery + chemotherapy | 1 |
| Chemotherapy + radiotherapy | 13 |

**Table 2** Toxicity of raltitrexed treatment, shown as worst WHO grade in 21 patients

| | WHO grade | | | | |
|---|---|---|---|---|---|
| | 0 | 1 | 2 | 3 | 4 |
| Leucopenia | 16 | 1 | 0 | 2 | 2 |
| Anaemia | 19 | 0 | 1 | 0 | 1 |
| Thrombocytopenia | 20 | 0 | 0 | 0 | 1 |
| Haemorrhage | 18 | 1 | 1 | 0 | 1 |
| Nausea and vomiting | 12 | 5 | 2 | 2 | 0 |
| Mucositis | 18 | 1 | 2 | 0 | 0 |
| Diarrhoea | 18 | 0 | 3 | 0 | 0 |

### Raltitrexed treatment and toxicity

Forty-one cycles of raltitrexed treatment were given (median two cycles, range 1–4). No dose reductions or delays occurred. The principal toxicities are shown in Table 2. The most commonly reported side-effects of raltitrexed treatment were nausea and asthenia (five patients), although vomiting was reported by only four patients. Mucositis, diarrhoea and reversible increases in liver transaminases were also reported. There were no episodes of neutropenic sepsis. Four patients died within 3 weeks of receiving raltitrexed. In only one of these cases was a drug-related adverse event (thrombocytopenia) implicated. The other deaths were attributed to progressive cancer.

### Responses

Among the 21 patients, no objective response was documented, three patients had stable disease, 16 had progressive disease and two died of disease. The median time to progression was 6 weeks (range 1–10 weeks). Ten patients went on to receive further cytotoxic chemotherapy. Of these, one achieved a PR, three had stable disease and five had progressive disease on their next treatment regimen. The median survival from study entry was 15 weeks (range 2–126 weeks).

### DISCUSSION

This phase II study indicates that raltitrexed given in this schedule is inactive in advanced small-cell lung cancer (96% certainty that the response rate is < 20%). The difficulties of assessing new drugs in tumours, such as small-cell lung cancer, that rapidly develop drug resistance have been widely debated (Cullen, 1989; Ettinger, 1990; Moore and Korn, 1992). Most investigators now favour testing new drugs for small-cell lung cancer in chemonaive patients with extensive disease or those who have relapsed after a previous response to chemotherapy. This was the group selected for inclusion in this study. Despite this, these patients had chemoresistant disease as indicated by a single disease response to subsequent conventional cytotoxic agents. The time to progression and overall survival are those expected for such patients.

Raltitrexed was well tolerated in this study. In previous studies, diarrhoea had been troublesome (Clarke et al, 1996). Here, no WHO grade 3 or 4 diarrhoea was reported, and no dose modifications were required. Only one patient died with persistent thrombocytopenia. This 51-year-old man had completed cranial and thoracic irradiation, and chemotherapy with cisplatin, ifosfamide and etoposide 10 weeks before study entry. He developed thrombocytopenia (platelets 24 $\times 10^9$ $l^{-1}$) and moderate leucopenia (WBC 1.9 $\times$ $10^9$ $l^{-1}$) 15 days after a second raltitrexed dose. The leucopenia recovered, but he was persistently thrombocytopenic at the time of his death from small-cell lung cancer on day 34.

Raltitrexed has shown promising activity in colorectal and breast cancers. We conclude that it is not effective as a second-line agent in advanced small-cell lung cancer. From these data, it would be difficult to justify a further study in chemonaive patients with extensive-stage small-cell lung cancer.

### REFERENCES

Clarke SJ, Hanwell J, de Boer M, Planting A, Verweij J, Walker M, Smith R, Jackman A, Hughes LR, Harrap KR, Kennealey GT and Judson IR (1996) Phase I trial of ZD1694, a new folate-based thymidylate synthase inhibitor, in patients with solid tumors. *J Clin Oncol* **14**: 1495–1503

Cullen M (1989) The design of phase II trials. *Lung Cancer* **5**: 214–220

Cunningham D, Zalcberg J, Rath U, Olver I, Van Cutsem E, Svensson C, Seitz JF, Harper P, Kerr D, Perez-Manga G, Azab M, Seymour L, Lowery K and the 'Tomudex' Colorectal Cancer Study Group (1995) 'Tomudex' (ZD1694): results of a randomised trial in advanced colorectal cancer demonstrate efficacy and reduced mucositis and leucopenia. *Eur J Cancer* **31A**: 1945–1954

Cunningham D, Zalcberg J, Smith I, Gore M, Pazdur R, Burris H, Meropol NJ, Kennealey G, Seymour L and the 'Tomudex' International Study Group (1996) 'Tomudex' (ZD1694): a novel thymidylate synthase inhibitor with clinical antitumour activity in a range of solid tumours. *Ann Oncol* **7**: 179–182

Ettinger DS (1990) Evaluation of new drugs in untreated patients with small cell lung cancer – its time has come. *J Clin Oncol* **8**: 374–377

Jackman AL, Taylor GA, Gibson W, Kimbell R, Brown M, Calvert H, Judson IR and Hughes LR (1991) ICI D1694, a quinazoline antifolate thymidylate synthase inhibitor that is a potent inhibitor of L1210 tumor cell growth in vitro and in vivo: a new agent for clinical study. *Cancer Res* **51**: 5579–5586

Moore TD and Korn EL (1992) Phase II trial design considerations for small cell lung cancer. *J Natl Cancer Inst* **84**: 150–154

Parker SL, Tong T, Bolden S and Wingo PA (1996) Cancer statistics, 1996. *CA Cancer J Clin* **65**: 5–27

Sorensen JM, Jordan E, Grem JL et al (1994) Phase I trial of the new thymidylate synthase inhibitor 'Tomudex' (ZD1694) in patients with advanced malignancy (abstract 240). *Ann Oncol* **5** (suppl. 5): 132

© Cancer Research Campaign 1997